# Identification of a DNA methylation signature in blood cells from persons with Down Syndrome

**DOI:** 10.18632/aging.100715

**Published:** 2014-01-08

**Authors:** Maria Giulia Bacalini, Davide Gentilini, Alessio Boattini, Enrico Giampieri, Chiara Pirazzini, Cristina Giuliani, Elisa Fontanesi, Maria Scurti, Daniel Remondini, Miriam Capri, Guido Cocchi, Alessandro Ghezzo, Alberto Del Rio, Donata Luiselli, Giovanni Vitale, Daniela Mari, Gastone Castellani, Mario Fraga, Anna Maria Di Blasio, Stefano Salvioli, Claudio Franceschi, Paolo Garagnani

**Affiliations:** ^1^ Department of Experimental, Diagnostic and Specialty Medicine, Alma Mater Studiorum - University of Bologna, Bologna 40138, Italy; ^2^ Interdepartmental Center “L. Galvani”, University of Bologna, Bologna 40126, Italy; ^3^ Personal Genomics S.r.l., Verona 37134, Italy; ^4^ Centro di Ricerche e Tecnologie Biomediche, Istituto Auxologico Italiano IRCCS, Via Zucchi 18, Cusano Milanino, 20095 Milan, Italy; ^5^ Department of Biological, Geological and Environmental Sciences, University of Bologna, Bologna 40126, Italy; ^6^ Department of Physics and Astronomy, University of Bologna, Bologna 40126, Italy; ^7^ Department of Medical and Surgical Sciences - Neonatology and Neonatal Intensive Care Unit, University of Bologna, Italy; ^8^ Institute of Organic Synthesis and Photoreactivity (ISOF) National Research Council (CNR), Bologna 40126, Italy; ^9^ Department of Clinical Sciences and Community Health, University of Milan, Milan, Italy; ^10^ Geriatric Unit, IRCCS Ca' Granda Foundation Maggiore Policlinico Hospital, Milan, Italy; ^11^ Cancer Epigenetics Laboratory, Instituto Universitario de Oncología del Principado de Asturias (IUOPA), HUCA, Universidad de Oviedo, Oviedo, Spain; ^12^ Department of Immunology & Oncology, Centro Nacional de Biotecnología/CNB-CSIC, Cantoblanco, Madrid, Spain; ^13^ IRCCS Institute of Neurological Sciences, Bologna, Italy; ^14^ Applied Biomedical Research Center, S. Orsola-Malpighi Polyclinic, Bologna 40138, Italy

**Keywords:** Down Syndrome, DNA methylation, Infinium Human Methylation 450 BeadChip, epigenetics, aging

## Abstract

Down Syndrome (DS) is characterized by a wide spectrum of clinical signs, which include segmental premature aging of central nervous and immune systems. Although it is well established that the causative defect of DS is the trisomy of chromosome 21, the molecular bases of its phenotype are still largely unknown. We used the Infinium HumanMethylation450 BeadChip to investigate DNA methylation patterns in whole blood from 29 DS persons, using their relatives (mothers and unaffected siblings) as controls. This family-based model allowed us to monitor possible confounding effects on DNA methylation patterns deriving from genetic and environmental factors. Although differentially methylated regions (DMRs) displayed a genome-wide distribution, they were enriched on chromosome 21. DMRs mapped in genes involved in developmental functions, including embryonic development (*HOXA* family) and haematological (*RUNX1* and *EBF4*) and neuronal (*NCAM1*) development. Moreover, genes involved in the regulation of chromatin structure (*PRMD8, KDM2B, TET1*) showed altered methylation. The data also showed that several pathways are affected in DS, including PI3K-Akt signaling. In conclusion, we identified an epigenetic signature of DS that sustains a link between developmental defects and disease phenotype, including segmental premature aging.

## INTRODUCTION

Down Syndrome (DS) results from the presence of all or part of an extra copy of human chromosome 21 (HSA21) [1] and it is the most common aneuploidy in humans, with about 1 in 700 live births [2]. The disease presents a broad range of clinical signs, which vary among individuals and include cognitive disorders, physical growth defects, endocrine disorders with a marked susceptibility to diabetes mellitus [3], diseases of the respiratory system and different cancer types [4].

DS is traditionally classified as a progeroid disease [5, 6], as affected subjects exhibit precocious appearance of age-associated biomarkers like DNA damage accumulation and chromosomal instability [7–10]. However, as recently reviewed by Zigman [11], accelerated aging in DS is atypical and segmental, involving some but not all organs and tissues, in particular the central nervous system [12–14] and the immune system [15, 16]. Accordingly, persons with DS suffer an accelerated decline of cognitive functions [14] and develop Alzheimer's disease with high frequency [11]. Moreover DS persons present peculiar haemato-logical abnormalities, that include abnormal platelet counts, macrocytosis, alterations in lymphocytes composition [17, 18] and higher susceptibility to develop leukemia, including rare forms like acute megakaryoblastic leukemia, and other hematopoietic disorders [19–21].

The molecular bases of DS pathogenesis are still largely unknown. Transcriptomic studies using microarrays and, more recently, next generation sequencing approaches have depicted a complex picture, in which altered RNA expression is detected for many but not all HSA21 genes and for an elevated number of non-HSA21 genes [22]. Recently, four independent studies started to dissect the epigenetic characteristics of DS, describing the DNA methylation patterns of different tissues at the genome wide level [23–25]. The first two studies used the Illumina Infinium Human-Methylation27 BeadChip (Infinium 27k) to analyze the DNA methylation profiles of chorionic villi samples [23] and of total peripheral blood leukocytes (PBL) and T-lymphocytes from adults with DS [25]. More recently, DNA methylation of DS placenta and buccal epithelium was assessed by reduced representation bisulfite sequencing and Illumina Infiunium HumanMethylation450 BeadChip (Infinium 450k) respectively [24, 26]. The studies were concordant in showing marked DNA methylation alterations in DS cells that were not enriched in HSA21, but were spread across the entire genome.

Here we used the Infinium 450k to analyze whole blood samples from a family-based model of DS. We used an analysis pipeline specifically tailored for Infinium 450k data, decribed in Bacalini *et al*.. Our model was composed by 29 persons with DS, their mothers and their unaffected siblings. This family-based model allowed us to monitor possible confounding effects on DNA methylation patterns deriving from genetic and environmental (lifestyle) factors. In addition, compared to the previous study on blood cells from DS [25] that used the Infinium 27k array, the Infinium 450k allows a deeper investigation of DNA methylation profiles, with a particular focus on CpG islands and their surrounding regions (shores and shelves) that are well established targets of differential methylation [27].

## RESULTS

### DNA methylation profile of persons with Down Syndrome

We used the Infinium 450k assay [28] to investigate the DNA methylation profile of peripheral white blood cells (WBC) from 29 trios composed by a DS person (DSP), the mother (DSM) and one non-affected sib (DSS). After quality check and the exclusion of chromosome X and Y data, we recovered 450981 out 485577 loci for subsequent analyses.

**Figure 1:**
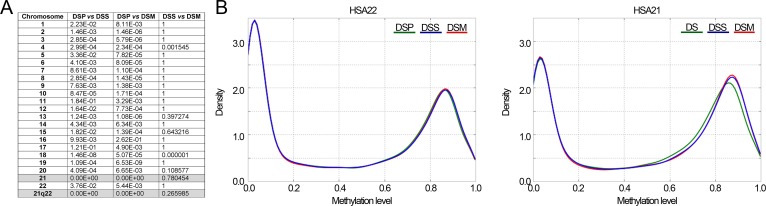
Comparison of DNA methylation distributions in DSP, DSS and DSM (**A**) Results of Kolmogorov-Smirnov test comparing, for each chromosome, the methylation distributions between DSP, DSS and DSM. The reported *p*-values are Bonferroni corrected. (**B**) Probability density distributions of methylation values in HSA22 (8179 CpG probes) and in HSA21 (4055 CpG probes).

To provide a global overview of the DNA methylation patterns of DS, we first compared the beta-values distributions of each chromosome between DSP, DSS and DSM. Significant differences between DSP and their relatives were observed for most chromosomes (Fig.1A). The most striking differences were observed in HSA21 (Fig.1A and Fig.1B), where DSP showed a decrease in the density of highly methylated loci with a concomitant enrichment in loci with methylation levels between 0.5 and 0.8.

### Cells count inference from DNA methylation data

Several studies reported age-dependent defects in the innate and in the adaptive immune system of persons with DS [29], consisting in altered prevalence of the different lymphocyte subpopulations. Considering that DNA methylation is tissue and cell specific, these differences could bias the discovery of differentially methylated regions (DMRs) when comparing DNA methylation of WBC from DSP with non-trisomic subjects. We reasoned that, in order to identify intrinsic DNA methylation defects in WBC from DSP, correction for lymphocyte subpopulations prevalence should be implemented in statistical analysis. However, in our cohort blood cell type counts were available only for DSP. To overcome this lack of information we took advantage of a recently developed algorithm to infer blood cell counts starting from DNA methylation data [30] that has been successfully applied in a study on rheumatoid arthritis [31]. The details of the cell counts estimation procedure are reported in Material and Methods section. The inferred cell counts faithfully reproduced our experimental data on DSP and the characteristic alterations in specific leukocytes populations (Supplementary Fig.1). In particular, DSP showed a significant decrease in the number of CD19+ B cells (*p*-value <0.001) and CD3+CD4+ T cells (*p*-value < 0.001) and a significant increase in the number of CD3+CD8+ T cells (*p*-value < 0.001), leading to altered CD3+CD4+/CD3+CD8+ ratio, as previously reported [15, 17]. Based on these considerations, we used inferred WBC counts as covariates in downstream analyses.

### Identification of differentially methylated regions between DSP and DSS

To identify DMRs between DSP and DSS we used an original analysis pipeline described by Bacalini *et al*.. Briefly, this approach classifies Infinium 450k probes in 4 Classes: *i)* Class A: probes in CpG islands and CpG islands-surrounding sequences (shores and shelves) that map in genic regions; *ii)* Class B: probes in CpG islands and CpG islands-surrounding regions (shores and shelves) which do not map in genic regions; *iii)* Class C: probes in genic regions which are not CpG rich; *iv)* Class D: probes in non-genic regions which are not CpG rich. Class A and Class B CpG probes were grouped in clusters, referred as “blocks of probes” (BOPs), containing the probes localized in the same island, in the same shore or in the same shelf. While Class C and Class D probes were compared between DS and DSS using ANOVA, Class A and Class B BOPs were compared using a multivariate analysis of variance (MANOVA), which allows to identify general changes in methylation of a genomic region.

**Figure 2:**
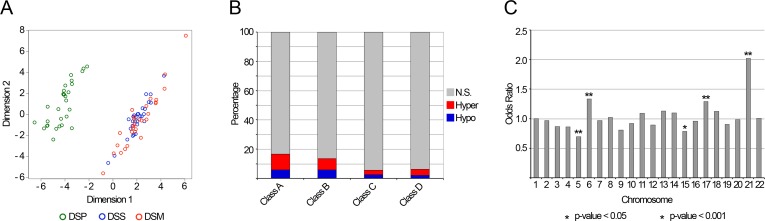
Down Syndrome associated DMRs (**A**) The MDS plot reports a bi-dimensional representation of the epigenetic distances between the samples under analysis, calculated using the methylation values of the 4648 BOPs selected as differentially methylated between DSP and DSS. (**B**) The percentage of identified DMRs is indicated for each of the four probes classes. DMRs are distinguished between hypermethylated and hypomethylated in DSP compared to DSS. (**C**) Chromosomal enrichment of the identified DMRs. For each chromosome, the Odds Ratio resulting from Fisher's exact test is reported. Significant enrichments are indicated with asterisks.

Sex, batch and cell types distribution were included in the analysis as covariates. In Class A, we identified 4648 BOPs able to discriminate DSP from DSS (*q*-value < 0.05; Fig.2A and Fig.2B). DSM methylation patterns were similar to those of DSS, confirming that the identified BOPs are specific of DS. It is worthwhile to note that the use of cell counts markedly reduced the number of identified DMRs (Supplementary Fig.2). Supplementary Table 1 reports the 4648 differentially methylated BOPs ranked on the basis of their *q*-value. Of the 6650 BOPs in Class B, 889 resulted differentially methylated between DSP and DSS (*q*-value < 0.05; Fig.2B and Supplementary Table 1).

Methylation values of Class C and Class D probes were compared between DSP and DSS by ANOVA, correcting for sex, batch and cell types distribution. 6051 CpG probes out of 109617 probes mapping in Class C and 3426 out of 54697 of probes mapping in Class D were differentially methylated between DSP and DSS (*q*-value < 0.05; Fig.2B and Supplementary Table 1).

Kerkel and co-workers previously analyzed DNA methylation profiles in peripheral blood lymphocytes (PBL) from 29 DS adults and 20 age-matched controls using the Infinium 27k and reported a list of differentially methylated genes distributed across various autosomes, with no specific enrichment on HSA21. Since most of the Infinium 27k probes are also in the Infinium 450k array, we checked whether the DMRs identified in that previous study were confirmed in our cohort. Among the 7 probes selected as DMRs by Kerkel *et al*. (cg07991621, cg08822227, cg09554443, cg05590257, cg14972143, cg00983520, cg21053323) all but one (cg05590257) resulted differentially methylated also in our cohort (Supplementary Fig.3). However, although it did not reach statistical significance, a trend for differential methylation was evident also for cg05590257 and the surrounding CpG probes.

When we looked at the distribution of Class A DMRs across the chromosomes, we found a significant overrepresentation of DMRs on chromosomes 21, 17, 6 and 15 and a significant underrepresentation on chromosome 5 (Fisher's exact test, *p*-value < 0.05; Fig. 2C). Chromosome 21 enrichment was observed also for CpG probes in Class C, but not for those in Class B and Class D (Additional File 8, Figure S7). While in chromosome 17 the DMRs were scattered along the entire chromosome, in chromosome 6 they clustered in the HLA locus. As this locus is highly polymorphic, the observed DNA methylation differences between DSP and DSS could be ascribed to the presence of SNPs in the probes of the array. To assess this point, we clustered the samples on the basis of their methylation values in the selected HLA loci, reasoning that the member of the same family should share some variants and for that should cluster together (Supplementary Fig.4). However, DSP tended to cluster together, suggesting that, at least for some HLA loci, their methylation profile was different from their relatives, independently from the genetic background.

Although HSA21 was the most affected by the aneuploidy, also almost all the other chromosomes were altered in terms of DNA methylation patterns. These results are consistent with those previously achieved with the 27k array, which however failed to identify the enrichment on chromosome 21, probably because of the lower density of probes, especially in small chromosomes with low number of genes.

### Pathway and gene ontology analyses

We performed Kyoto Encyclopedia of Genes and Genomes (KEGG) pathway and gene ontology (GO) analyses using the list of 4648 Class A BOPs differentially methylated between DS and DSS (Table 1). The KEGG pathways that reached statistical significance after FDR correction were involved in ribosome, immune functions and type I diabetes. GO analysis revealed a number of enriched GO terms, with the selected DMRs predominantly mapping in genes involved in developmental processes and in the morphogenesis of anatomical structures.

**Table 1:** KEGG pathways and gene ontology analysis for Down Syndrome associated DMRs. The table reports the significantly enriched KEGG pathways and gene ontologies, as resulting from the analysis with Fisher's exact test and GOrilla platform (see Materials and methods section)

Description	q-value
*Kegg Pathway*	
Ribosome	0.013
Allograft rejection	0.013
Graft-versus-host disease	0.013
Cell adhesion molecules (CAMs)	0.013
Autoimmune thyroid disease	0.013
PI3K-Akt signaling pathway	0.013
Basal cell carcinoma	0.013
HTLV-I infection	0.034
Type I diabetes mellitus	0.040
*Gene Ontology Process*	
System process (GO:0003008 )	0.027
Anatomical structure morphogenesis (GO:0009653 )	0.032
Regulation of signal transduction (GO:0009966 )	0.027
Multicellular organismal process (GO:0032501 )	0.000
Single-organism process (GO:0044699 )	0.015
Single-multicellular organism process (GO:0044707 )	0.000
Positive regulation of biological process (GO:0048518 )	0.027
Embryonic organ morphogenesis (GO:0048562 )	0.006
Regulation of response to stimulus (GO:0048583 )	0.035
Embryonic skeletal system morphogenesis (GO:0048704 )	0.018
Anatomical structure development (GO:0048856 )	0.017
Regulation of body fluid levels (GO:0050878 )	0.038

### Identification of an epigenetic signature of Down Syndrome

**Figure 3:**
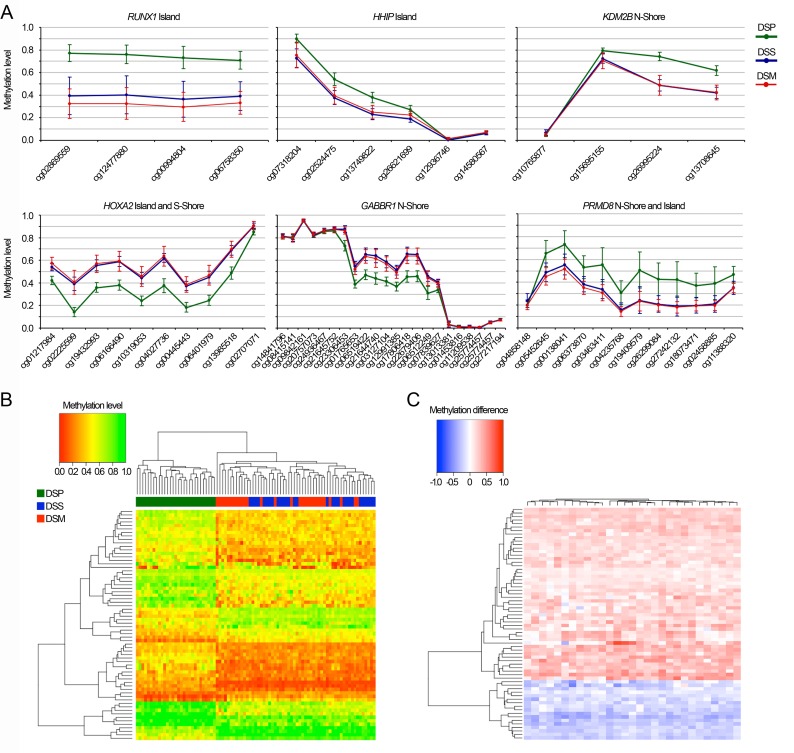
Epigenetic signature of Down Syndrome (**A**) DNA methylation profiles of 6 of the 68 BOPs included in the epigenetic signature of DS. (**B**) The heatmap reports DNA methylation values for the 68 BOPs included in the epigenetic signature of DS (CpG probes in rows, samples in columns and color-coded). Dendrograms depicts hierarchical clustering of probes and samples. (**C**) For the 68 BOPs included in the epigenetic signature of DS, the heatmap reports DNA methylation differences between each DSP and his/her DSS (CpG probes in rows, samples in columns). Dendrograms depicts hierarchical clustering of probes and samples (DSP-DSS pairs). Both in (**B**) and in (**C**) the methylation value of the most significant CpG probe within each BOP was considered.

To provide an unambiguous epigenetic signature of DS, from the list of 4648 Class A BOPs altered in DSP we selected a short list of DMRs whose DNA methylation status was remarkably different compared to healthy sibs. To this aim, we considered only the BOPs containing at least 2 adjacent CpG sites for which the DNA methylation difference between DSP and DSS was higher than 0.15, as previously suggested [32]. Of the 4648 BOPs selected above, 68 met these more stringent criteria (Supplementary Table 2). Fig. 3A reports the DNA methylation profile for some of the selected BOPs. Hierarchical clustering analysis showed that the methylation status of the 68 loci clearly separated DSP from DSS and DSM, while it did not distinguish DSS from DSM (Fig. 3B). 73% of the probes included in this epigenetic signature were hypermethylated in DSP respect to DSS.

To investigate if our selection of CpG probes universally characterizes DS, independently from genetic or environmental factors, we took advantage of our family-based cohort and we calculated for each DSP-DSS pair the difference between the methylation levels of the most significant CpG probe in each of the 68 BOPs. Hierarchical clustering of the difference values did not clearly distinguish any family from the others, indicating that the identified signature is not significantly affected by genetic or environmental factors (Fig. 3C).

The number of loci included in the epigenetic signature of DS was too small to perform ontology enrichment analyses, however from a careful screening of the list four main functions emerged: 1) haematopoiesis (*RUNX1, DLL1, EBF4* and *PRMD16*); 2) morphogenesis and development (*HOXA2, HOXA4, HOXA5, HOXA6, HHIP, NCAM1*); 3) neuronal development (*NAV1, EBF4, PRDM8, NCAM1, GABBR1*); 4) regulation of chromatin structure (*PRMD8, KDM2B, TET1*).

**Figure 4:**
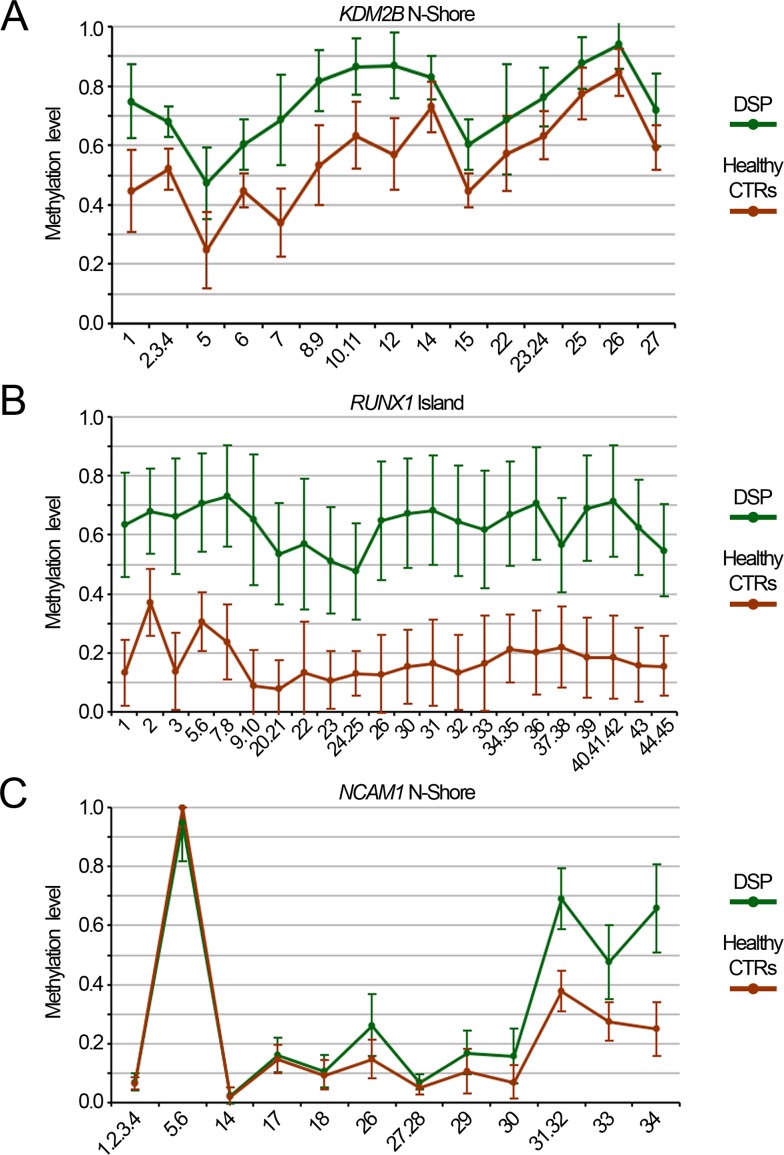
Validation of Down Syndrome DMRs by Sequenom EpiTYPER DNA methylation levels of the N-Shore of *KDM2B* (**A**), the island of *RUNX1* (**B**) and the island of *NCAM1* (**C**) assessed in whole blood from a cohort of 78 DS and 62 age- and sex-matched healthy controls.

Finally, we validated 3 of the DMRs included in the epigenetic signature of DS (*RUNX1* island, *KDM2B* N-Shore and *NCAM1* island) using an alternative method, the Sequenom's EpiTYPER assay. Besides the 29 DSP and 29 DSS used for genome wide DNA methylation analysis, the validation cohort included additional 49 DSP and 33 age- and sex- matched unrelated controls. EpiTYPER analysis confirmed that the CpG sites included in the 450k BeadChip were differentially methylated between DSP and controls and showed that the DMRs extended also to the adjacent CpG sites. In particular, in *RUNX1* and *KDM2B* amplicons all the CpG sites resulted significantly hypermethylated in DSP respect to controls (Fig. 4A and Fig. 4B; Student's t-test). On the contrary, only 7/11 of the CpGs assessed in *NCAM1* island were significantly different between DSP and controls (Fig. 4C). As DS can be characterized by total or partial trisomy, we checked whether this could affect the methylation of these DMRs. No significant difference between free trisomy and translocation or mosaicism was found (data not shown).

## DISCUSSION

In this study we investigated the epigenetic status, in terms of DNA methylation, of WBC from DS persons (DSP), in comparison with their mothers (DSM) and unaffected sibs (DSS). This family-based model was chosen in order to minimize confounding genetic and environmental factors.

Our analysis showed that DS is characterized by a profound rearrangement of genome-wide DNA methylation patterns. Overall distributions of DNA methylation values resulted significantly different between DSP and their unaffected sibs (and mothers) for most chromosomes, but these differences were particularly marked for HSA21. Accordingly, region-centric and site-specific analyses identified a large number of DMRs that, although mapping to all chromosomes, were enriched in HSA21 for Class A and Class C probes. This result is particularly interesting, as the probes in these two classes are associated to genic sequences (Bacalini *et al*.). As the Infinium 450k provides an estimate of the mean methylation status of the DNA hybridized to the array, it is difficult to say if only the extra copy of HSA21 is aberrantly methylated or if DNA methylation changes affect all the 3 copies of HSA21. Future studies should address this point by analyzing allele-specific DNA methylation. DMRs enrichment in HSA21 was not observed in the three studies that have previously analyzed DNA methylation patterns in DS, but this discrepancy could be ascribed to the different experimental procedures, analytical protocols, analyzed tissues and/or age classes of the probands. However, it is worth to note that our analysis confirmed the majority of DMRs previously identified in PBL from DS [25].

We observed a prevalent hypermethylation of DS DMRs, although less pronounced than the one described in placental tissue [24]. Interestingly, Jin and coworkers have proposed that these hypermethylation events occur early during development as a consequence of downregulation of TET enzymes and/or of *REST* transcription factor. In our dataset, *REST* N-shore resulted slightly hypermethylated in DSP, potentially confirming downregulation of *REST* mRNA in DS, while *TET1* S-shore was hypomethylated. To investigate the reasons of this discrepancy, it would be interesting to experimentally verify if *TET1* altered expression in DSP is tissue-dependent and how DNA methylation could regulate its transcription.

Starting from the list of Class A DMRs, we defined an epigenetic signature of DS in WBC by selecting a short list of DMRs that show a methylation difference between DSP and DSS greater than 0.15 and that can be functionally linked to the various phenotypic aspects of the disease. Among the genes included in the signature there is *RUNX1*, which encodes for a transcription factor that is pivotal in the development of haematopoietic cells and that can be involved in acute myeloid leukaemia [33, 34], whose frequency is high among DSP. Another gene that could account for the haematological defects in DSP is *EBF4*, which belongs to a family of transcription factors involved in B-cell maturation [35]. Consistently, B lymphocytopenia is a main immunological characteristic of DS [15, 17, 36]. The signature includes also genes belonging to the *HOXA* cluster, whose fine tuning during development, also through epigenetic mechanisms, is a crucial determinant of embryonic cell fate [37]. Interestingly, we found that the selected DMRs mapped also to genes that are involved in the development of tissues other than the blood, *in primis* the central nervous system (CNS). Although the methylation profiles are highly tissue-specific, these alterations of DNA methylation in WBC can be clues of epigenetic defects in other tissues, such as the CNS, that can contribute to DS pathogenesis. Finally, our short list included genes involved in the regulation of chromatin [38], such as *TET1* and *KDM2B*, an histone lysine demethylase that in mouse embryonic stem cells recruits the polycomb repressive complex 1 (PRC1) to CpG islands of early lineage-specific genes [39, 40]. As *KDM2B* has also been shown to repress the expression of ribosomal genes, its hypermethylation could account for the increase in ribosomal gene activity previously observed in lymphocytes from persons with DS [41, 42]. Importantly, we showed that the selected DNA methylation changes were common to all the analyzed DSP, independently from their genetic and environmental background, and were reproducible in an independent cohort.

Our analysis supports the perspective of DS as a developmental disease [43], as many of the identified DMRs are involved in morphogenetic and developmental processes. This observation poses the basis for a link between intrinsic defects that are established early during development [29, 44, 45] and DS phenotype, including the precocious functional aging of specific tissues. Indeed, as described above, we identified genes with altered DNA methylation that are involved in the development of nervous and immune systems, which both show an aged phenotype in DS.

Also KEGG results provided interesting insights into the molecular basis of DS phenotype. PI3K-Akt signaling pathway regulates fundamental cellular functions, such as protein synthesis and cell proliferation, and has key roles in aging [46, 47]. Three independent groups have recently demonstrated that the PI3K/Akt/mTOR pathway is deregulated in DS and that this event occurs early in development [48–50]. Combining these observations with our results we can speculate that early alteration in methylation of genes involved in PI3K/Akt/mTOR can contribute to DS phenotype, including signs of premature aging. KEGG analyses sustain also a strong link between aneuploidy-linked DNA methylation changes and the higher prevalence of immunological disorders in persons with DS [29, 51]. In this sense it is worth to note that in DS the methylation profile of the HLA locus on chromosome 6 is heavily altered. Although we cannot exclude that a significant fraction of these differences between DSP and DSS can be ascribed to the highly polymorphic nature of the region and to inter-individual variations of its methylation profiles [52], that could have hampered our analysis, we are tempted to speculate that changes in DNA methylation could affect HLA regulation, thus contributing to immunological and autoimmune defects in DS.

Two main theories have been formulated in the last decades to unravel the molecular bases of DS pathogenesis [53]. According to the reductionistic theory, a “Down Syndrome critical region” (DSCR) on HSA21 [54] includes a limited number of dosage-sensitive genes whose trisomy results in the profound phenotypic alterations of DS. In this perspective, DS pathogenesis should map to a specific genetic regions of HSA21. As an alternative to the DSCR theory, the “organicist concept” centers the DS pathogenesis on the developmental process and suggests that the presence of a trisomic chromosome, beside the specific function of the genes that it contains, disrupts on the whole the genetic homeostasis and leads to developmental instability. Both murine [55, 56] and human models [57, 58] have recently argued against the existence of a unique DSCR, proposing that HSA21 contains different susceptibility regions that can contribute to the DS phenotype. Based on these and on other data, current researchers favour a synthesis of the two theories, in which the combination of the trisomy of multiple HSA21 genes, none of which is by itself critical for the disease, induces a wide-range cascade of events that through physical and functional interactions engages many non-HSA21 genes and results in a global remodeling of genomic function. Our results support this view, as we identified DS associated DMRs that, although enriched on HSA21, interest most of the other chromosomes and are functionally linked to the developmental defects characteristic of the disease. Future studies should investigate the origin of the observed DNA methylation defects in DS. A link between alterations in DNA methylation patterns and genetic defects in genes with epigenetic functions has been established for many diseases, including immunodeficiency, centromeric regions Instability and Facial anomalies Syndrome (ICF), Rett Syndrome and cancer [59]. In the case of DS, this connection appears less clear. It is interesting to note that among the genes on HSA21 there is *DNMT3L*, which encodes for a protein that, although missing enzymatic activity, assists *de novo* DNA methyltransferases in establishing DNA methylation marks [60]. The trisomic status of *DNMT3L* could therefore affect the establishment of DNA methylation patterns during development. Moreover, while some methylation changes could be directly caused by alterations in signal transduction cascades due to gene-dosage imbalances, other DNA methylation variations could be caused by feedback mechanisms that try to buffer RNA expression defects in trisomic cells. Finally, changes in DNA methylation profiles could be ascribed also to the perturbation of the conserved but fine-tuned network of chromosome interactions that rules nuclear functions.

## METHODS

### Samples

Persons with DS (DSP) who participated to this study were part of a larger open-label study on cognitive decline in DS described elsewhere (Ghezzo et al., 2014). The study was approved by the local Ethical Committee (S. Orsola Hospital, University of Bologna; ethical clearance #126/2007/U/Tess, released on December 18, 2007). Written informed consent to participate in the study was obtained from adult DS persons and from parents or authorised tutors for those under age. Written informed consent was also obtained for adult DS persons from parents or relatives (brothers/sisters). Subjects were recruited with the help of CEPS, OPIMM and ANFFAS, three local non-profit associations dealing with DS persons operating in the eastern part of Emilia-Romagna Region (Bologna and Ferrara provinces). Participation in the study was on a totally voluntary basis, with no reward for the participants or their families. Exclusion criteria were current acute illnesses, hepatic, renal or cardiac insufficiency, assumption of antioxidant or nutraceutical substances (vitamins, lipoic acid, acetylcysteine, omega 3 and 6 fatty acids, probiotics) within the last two months, as detailed elsewhere [14] A total of 29 DSP (12–43 years, 18 males, 11 females), 29 DSS (9–52 years, 7 males, 22 females) and 29 DSM (42–83 years) were included in the study. All the DSP were classified as free trisomy with the exception of 3 translocations and 4 mosaicisms.

### DNA extraction and bisulphite treatment of DNA

Extraction of genomic DNA from whole peripheral blood was performed using the QIAamp 96 DNA Blood Kit (QIAGEN, Hilden, Germany). Sodium bisulphite conversion for Infinium HumanMethylation450 BeadChip and for Sequenom EpiTYPER assay was performed using the EZDNA Methylation-Gold Kit and the EZ-96 DNA Methylation Kit respectively, as previously described [61].

### Genome-wide DNA methylation analysis

Genome-wide DNA methylation of 29 families including a DSP, a DSM and a DSS was analyzed using the Infinium HumanMethylation450 BeadChip (Illumina, San Diego, CA) following manufacturer's instructions. Arrays were scanned by HiScan (Illumina). GenomeStudio (Illumina) was used to perform background subtraction, while IMA R package [62] was used to pre-process the *β*-values. All the samples were retained, as none had more than 75% of the probes with a detection *p*-value greater than 1e-05. 425 probes had a detection *p*-value greater than 0.05 in more than 75% samples and were removed, together with the probes containing missing values (23437) and those localized on sexual chromosomes. Based on these quality checks, 450981 out of 485577 CpG were retained.

### Statistical analysis

For each chromosome, beta-values distributions in DSP, DSS and DSM were reported as 100-bins histograms, that were compared using Kolmogorov-Smirnov test. Bonferroni correction was performed to correct for multiple testing.

The RELATION_TO_UCSC_CPG_ISLAND and the UCSC_REFGEN_NAME columns in the Illumina output were used to subset the array probes in four classes and to group probes in BOPs, as described in Bacalini et al.. Class A included 229232 probes grouped in 73442 BOPs, 30400 of which included 2 or more probes (176211 probes); Class B included 57370 probes grouped in 32176 BOPs, 6650 of which included 2 or more probes (28051 probes); Class C 109617 probes; Class D included 54762 probes.

For Class A and Class B, BOPs methylation values were compared between DS and DSS using the MANOVA function from the R package *car*. BOPs containing one or 2 CpG probes were excluded from the analysis and MANOVA was applied on sliding windows of 3 consecutive CpGs within the same BOP. For each BOP, we kept the lowest *p*-value among those calculated for the different sliding windows. For Class C and Class D, the methylation values of the probes were compared between DS and in DSS using the ANOVA function from the R package *car*. Both for MANOVA and ANOVA analysis, correction for sex, batch and cell counts (see the next paragraph) were performed. To correct for multiple testing, we applied a Benjamini-Hochberg False Discovery Rate correction using the function *mt.rawp2adjp* from the R package *multtest*. Gene ontology annotation of selected DMRs was performed using gene ontology enrichment analysis and visualization tool http://cbl-gorilla.cs.technion.ac.il/ [63]. For KEGG pathways, the significance analysis was performed using a two tailed Fisher's exact test for each pathway [64].

### Estimation of cell counts

We used a previously published algorithm [30] to infer white blood cell counts from DNA methylation data. Starting from cell-specific DNA methylation signatures of purified leukocyte samples (validation dataset), the method selects *n* CpG sites with the highest informativeness with respect to blood cell types and uses this information to predict leukocytes distribution in the target dataset. To our knowledge, two validation datasets are currently available in public databases. In the first dataset (GEO Accession number GSE39981), DNA methylation profiles of 46 samples (6 CD19+ B cells samples, 8 granulocytes samples, 5 CD14+ monocytes samples, 11 CD56+ NK cells samples, 8 CD3+CD4+T cells samples, 2 CD3+CD8+ T cells samples, 1 CD3+CD56+ NK sample and 5 CD3+ T cells samples) were analyzed by the Infinium 27k. The second dataset (GEO Accession number GSE35069) includes data from the Infinium 450k on seven sorted cell populations (CD4+ T cells, CD8+ T cells, CD56+ NK cells, CD19+ B cells, CD14+ monocytes, neutrophils, and eosinophils) from six healthy males. We tested the performance of both validation sets by comparing the predicted leukocytes distributions with flow cytometry results available for DS. Although both datasets were successful in predicting experimentally measured cell counts, we noticed that the GSE35069 was less effective in predicting specific cell types such as CD56+ NK cells (Supplementary Figure 2A-D). This difference persisted also when we tried to use different sets of informative CpG sites selected from the two validation datasets (100, 300, 500, 1000, 2000 CpG sites from the GSE39981 dataset; 100, 300, 500, 1000, 5000, 10000 and 20000 CpG sites from the GSE35069 dataset). Based on these considerations, we decided to use GSE39981 as validation dataset and we estimated the distribution of CD19+ B cells, CD3+CD4+ T cells, CD3+CD8+ T cells, granulocytes, CD14+ monocytes and CD56+ NK cells using the 500 most informative CpG sites, 453 of which were included in the Infinium 450k. Projections of cell types distributions for DS, DSS and DSM are reported in Supplementary Figure 2B.

### Locus-specific DNA methylation analysis

The EpiTYPER assay (Sequenom, San Diego, CA) was used for the quantitative analysis of DNA methylation of CpGs in *RUNX1* CpG island (chr21:36, 258, 992–36, 259, 453), *KDM2B* N-Shore (chr12:121, 973, 796–121, 974, 353) and *NCAM1* island (chr11:112, 834, 144–112, 834, 547). 10 ng of bisulphite-treated DNA were PCR-amplified and processed following manufacturer's instructions. Bisulphite specific primers were the following: *RUNX1*_Forward: aggaagagagGGTAGGAGTTGTTTGTAGGGTTTTAAT; *RUNX1*_Reverse: cagtaatacgactcactatagggagaaggctCCCACATCCCAAACTAAAAAAA; *KDM2B*_Forward: aggaagagagGGGATTTTGATTATTTTATTGTTAGTTT; *KDM2B*_ Reverse: cagtaatacgactcactatagggagaaggctAAAACCCCTCCCTACCACTTAC; *NCAM1*_Forward: aggaagagagGGGAGGGTATTTTGGTAGGTATATTT; *NCAM1*_Reverse: cagtaatacgactcactatagggagaaggctAAAATTCCTAAACCTACAACTTCCAC.

## SUPPLEMENTARY TABLES AND FIGURES






