# Supplementary material for: Identification of a DNA methylation signature in blood cells from persons with Down Syndrome
Source: Aging (Albany NY). 2014 Jan 8;7(2):82–93. doi: 10.18632/aging.100715 (PMC4359691; doi:10.18632/aging.100715)
Supplement: Supplementary file 3 [file aging-07-0082-s003.pdf]

SUPPLEMENTARY TABLES

Please follow the links in full text version of this paper to see the Supplementary Tables 1 and 2.

SUPPLEMENTARY FIGURES

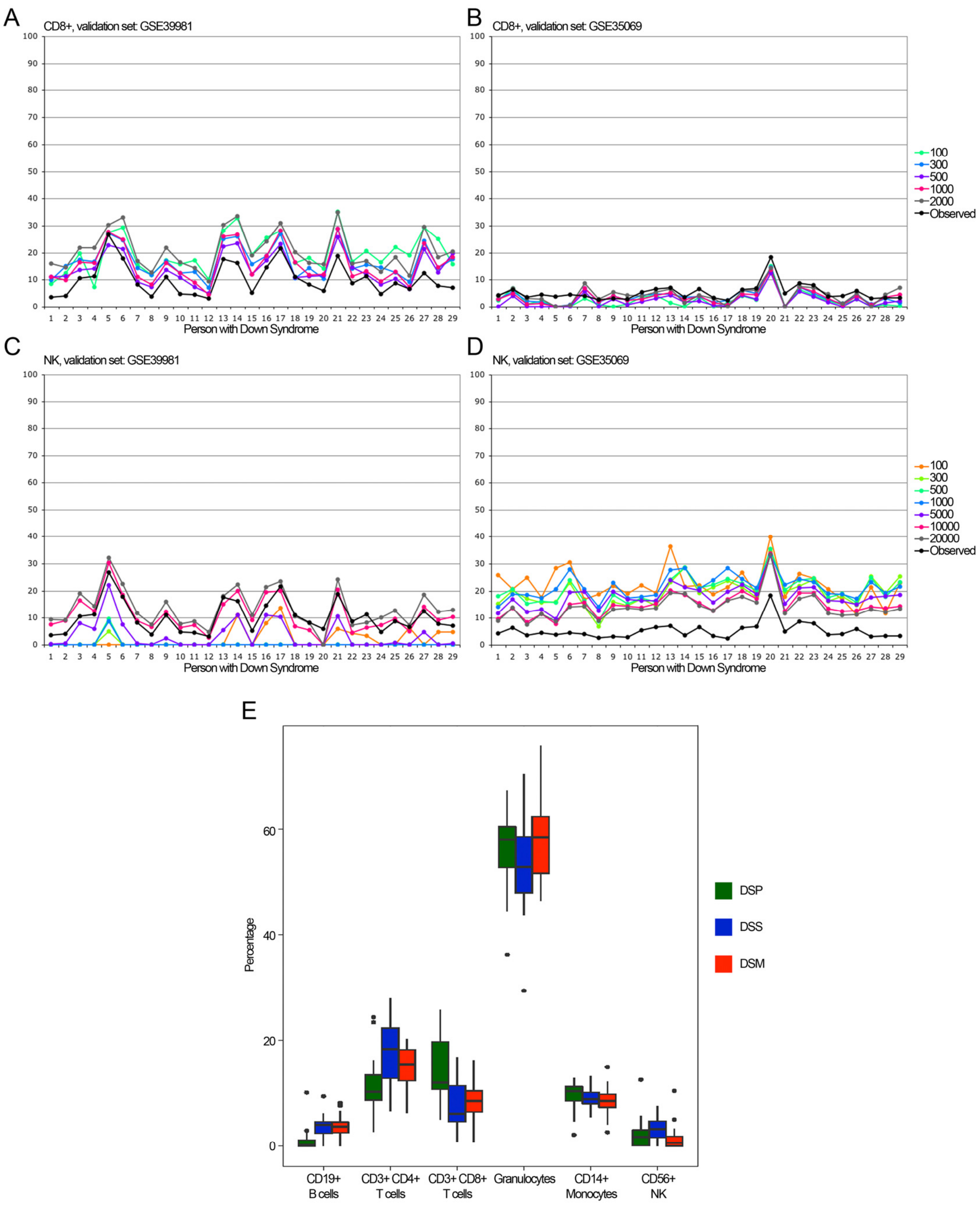

Supplementary Figure 1. Estimation of cell counts. (**A**) The four examples report the results of cell counts estimation for CD3+CD8+ T cells (upper panels) and CD56+ NK cells (lower panels), calculated using two different validation sets (GSE39981, left panels, and GSE35069, right panels) and different sets of informative CpG sites selected from the two validation datasets (100, 300, 500, 1000, 2000 CpG sites from the GSE39981 dataset; 100, 300, 500, 1000, 5000, 10000 and 20000 CpG sites from the GSE35069 dataset). The observed cell counts estimated by flow cytometry are reported in black. (**B**) Distributions of estimated cell counts for DSP, DSS and DSM.

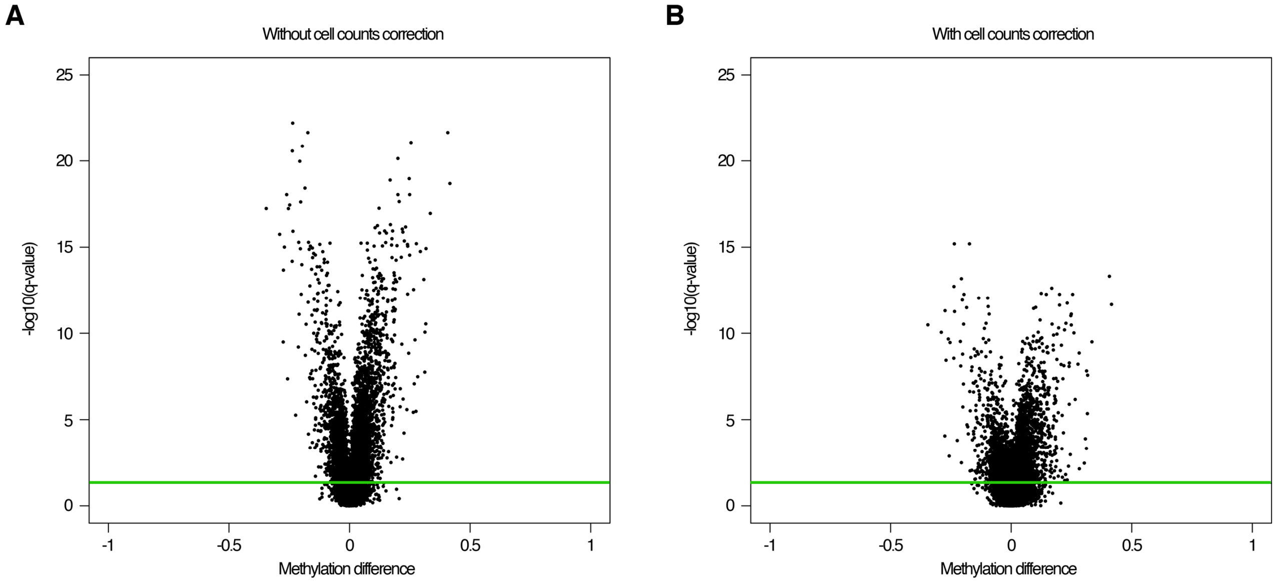

Supplementary Figure 2. Effect of adjustment for estimated cell counts. For each BOP, the CpG probe most significantly differentially methylated between DSP and DSS was selected. The volcano plot reports the $-\log_{10}$ (q-value) of each BOP against the methylation difference between DSP and DSS for the selected CpG probe, without (**A**) or with (**B**) correction for cell counts, in addition to correction for sex and batch effects. The green lines represent q-value=0.05.

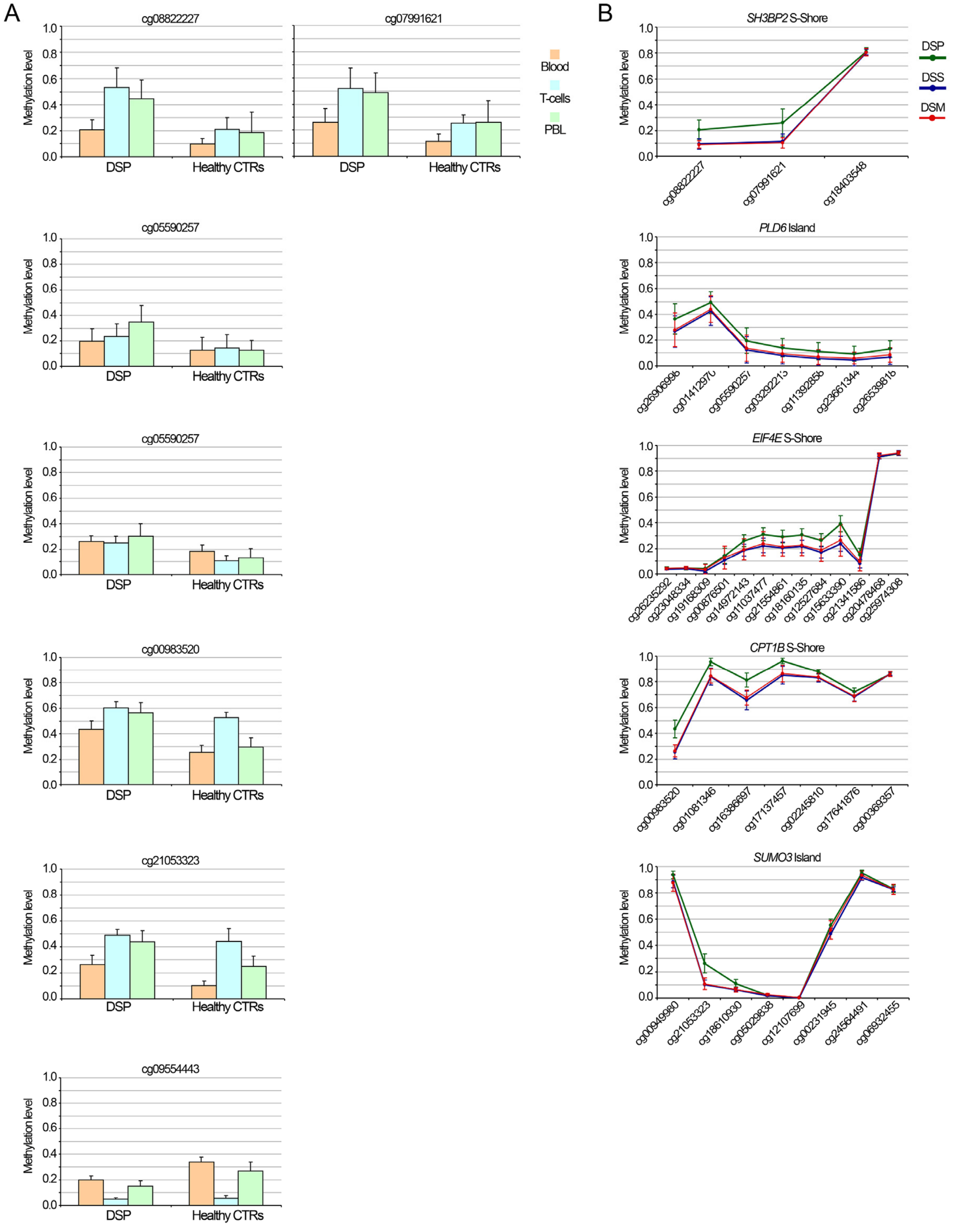

Supplementary Figure 3. Confirmation of previous studies. (**A**) The DNA methylation levels of the CpG probes previously selected as differentially methylated in DS compared to healthy controls in T-cells and in PBL (Kerkel). For the same probes the DNA methylation values in whole blood, measured in this study, are indicated. **(B)** Whole blood DNA methylation profile of the BOPs containing the CpG probes reported in (**A**). The probe cg09554443 maps to a non CpG rich region of the *CD247* gene and the corresponding BOP is not reported.

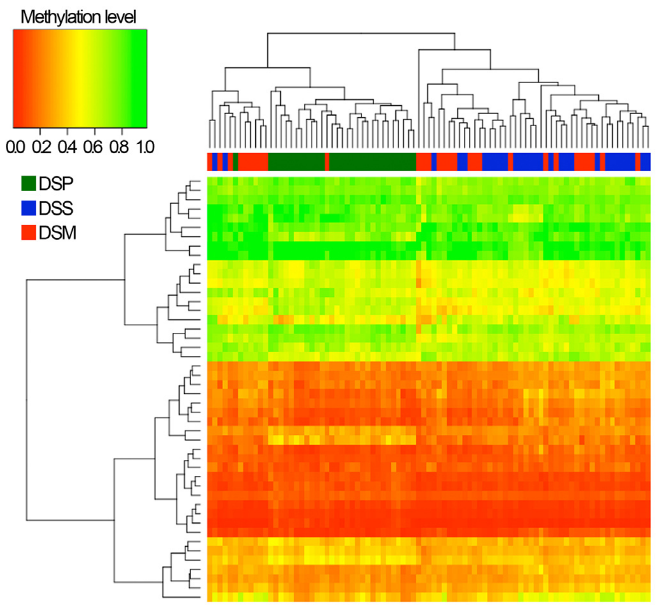

Supplementary Figure 4. Hierarchical clustering of DSP, DSS and DSM for significative BOPs mapping in HLA loci. For the 21 BOPs mapping in HLA loci, the CpG probes differentially methylated between DSP and DSS were selected (ANOVA, q-value <0.05; 46 CpG probes). The heatmap reports DNA methylation values for the selected CpG probes (CpG probes in rows, samples in columns and color-coded). Dendrograms depicts hierarchical clustering of probes and samples.

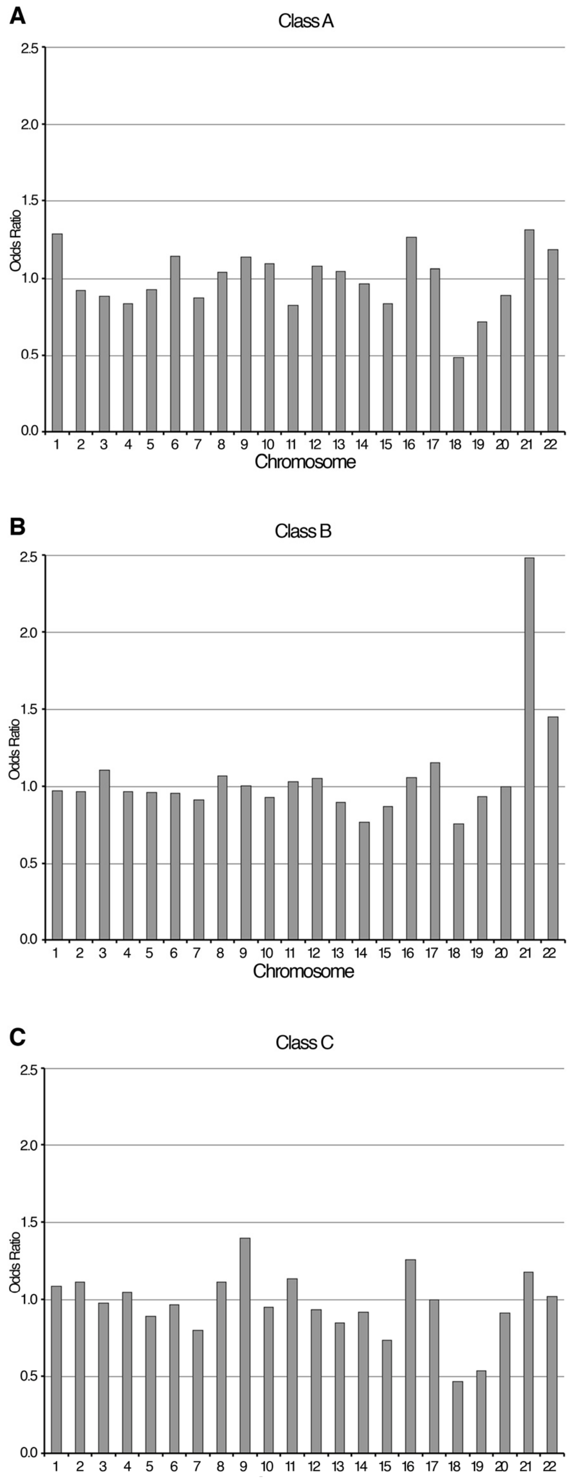

Supplementary Figure 5. Chromosomal enrichment of DMRs in Class B, Class C and Class D probes. For each chromosome, the Odds Ratio resulting from Fisher's exact test is reported. Significant enrichments are indicated with asterisks.
